# The accuracy of prehospital triage decisions in English trauma networks – a case-cohort study

**DOI:** 10.1186/s13049-024-01219-9

**Published:** 2024-05-21

**Authors:** G. Fuller, J. Baird, S. Keating, J. Miller, R. Pilbery, N. Kean, K. McKnee, J. Turner, F. Lecky, A. Edwards, A. Rosser, R. Fothergill, S. Black, F. Bell, M. Smyth, JE. Smith, GD. Perkins, E. Herbert, S. Walters, C. Cooper, the MATTS research group, Ian Maconochie, Mathew Ward, Mark Millins, Emily Turton, Simon Waterhouse, Matt Stevenson, Daniel Pollard, Abdullah Pandor, Maria Robinson, Stuart Reid, Di Charles

**Affiliations:** 1 School of Health and Related Research, University of Sheffield, Regent Court, Regent Street, Sheffield, S1 4DA UK; 2 CorEvitas, Waltham, USA; 3 West Midlands Ambulance Service, Brierley Hill, UK; 4 Yorkshire Ambulance Service, Wakefield, UK; 5 South Western Ambulance Service, Exeter, UK; 6 Trauma Audit and Research Network, Manchester, UK; 7 London Ambulance Service, London, UK; 8 The University of Warwick, Coventry, UK; 9 University Hospitals Plymouth NHS Trust, Plymouth, UK

**Keywords:** Major trauma, Injuries, Triage, Diagnostic accuracy, Triage tools, Case-cohort

## Abstract

**Background:**

Care for injured patients in England is provided by inclusive regional trauma networks. Ambulance services use triage tools to identify patients with major trauma who would benefit from expedited Major Trauma Centre (MTC) care. However, there has been no investigation of triage performance, despite its role in ensuring effective and efficient MTC care. This study aimed to investigate the accuracy of prehospital major trauma triage in representative English trauma networks.

**Methods:**

A diagnostic case-cohort study was performed between November 2019 and February 2020 in 4 English regional trauma networks as part of the Major Trauma Triage Study (MATTS). Consecutive patients with acute injury presenting to participating ambulance services were included, together with all reference standard positive cases, and matched to data from the English national major trauma database. The index test was prehospital provider triage decision making, with a positive result defined as patient transport with a pre-alert call to the MTC. The primary reference standard was a consensus definition of serious injury that would benefit from expedited major trauma centre care. Secondary analyses explored different reference standards and compared theoretical triage tool accuracy to real-life triage decisions.

**Results:**

The complete-case case-cohort sample consisted of 2,757 patients, including 959 primary reference standard positive patients. The prevalence of major trauma meeting the primary reference standard definition was 3.1% (*n*=54/1,722, 95% CI 2.3 – 4.0). Observed prehospital provider triage decisions demonstrated overall sensitivity of 46.7% (*n*=446/959, 95% CI 43.5-49.9) and specificity of 94.5% (*n*=1,703/1,798, 95% CI 93.4-95.6) for the primary reference standard. There was a clear trend of decreasing sensitivity and increasing specificity from younger to older age groups. Prehospital provider triage decisions commonly differed from the theoretical triage tool result, with ambulance service clinician judgement resulting in higher specificity.

**Conclusions:**

Prehospital decision making for injured patients in English trauma networks demonstrated high specificity and low sensitivity, consistent with the targets for cost-effective triage defined in previous economic evaluations. Actual triage decisions differed from theoretical triage tool results, with a decreasing sensitivity and increasing specificity from younger to older ages.

**Supplementary Information:**

The online version contains supplementary material available at 10.1186/s13049-024-01219-9.

## Introduction

The care of seriously injured patients in England was noted to be sub-optimal in a 2007 report by the National Confidential Enquiry into Patient Outcome and Death, where 60% of cases demonstrated deficiencies in organisational or clinical aspects of care [1]. A subsequent 2010 National Audit Office enquiry confirmed these findings, highlighting the ad hoc organisation of trauma care, with unacceptable variations in mortality rates, depending on where and when a patient received treatment [2].

In response, trauma care in England was reconfigured with the introduction of inclusive regional trauma networks in 2011 [3]. These systems of care consist of a central major trauma centre (MTC) hospital providing specialist resuscitation, definitive care, and rehabilitation to the highest acuity and most seriously injured patients. Non-specialist general hospitals, termed trauma units, manage less seriously injured patients and can provide stabilisation and transfer of the more seriously injured to MTCs when needed. Other acute hospitals, which would not routinely manage injured patients, are designated Local Emergency Hospitals. Management, training, and governance structures are incorporated to coordinate patient management and ensure high quality care is delivered [4].

English ambulance services use triage tools to identify patients with major trauma who would benefit from expedited MTC care. Developed from the American College of Surgeons Committee on Trauma Field Triage Guidelines, these consist of a checklist of physiological and injury variables [5]. Their primary purpose is to identify appropriate patients for direct transportation to MTCs, potentially bypassing other hospitals that may be closer to the location of injury. A second purpose, relevant to patients regardless of injury location, is to guide pre-alert calls to the MTC emergency department to allow activation of a hospital trauma team and rapid resuscitation. Triage tools must balance under- and over-triage, to ensure patients with major trauma are appropriately treated, but MTC resources are not wasted unnecessarily.

Introduction of trauma systems has been associated with improved outcomes for patients with major trauma [6]. However, there has been no investigation of triage tool performance, despite their pivotal role in ensuring effective and efficient MTC care. Each English ambulance service uses a different triage tool, varying in structure and content. This study aimed to characterise major trauma triage tool performance in representative English trauma networks. Specific objectives were to describe the characteristics of patients presenting to ambulance services with non-trivial injury, to evaluate the accuracy of real-life triage decisions, and to investigate the theoretical accuracy of triage tools.

## Methods

### Study design

A diagnostic case-cohort study was undertaken to evaluate the accuracy of major trauma triage in representative English trauma networks [7]. Study procedures and reporting followed the principles stated in STARD guidelines for performing diagnostic accuracy studies [8]. This study was conducted as a planned secondary analysis of the Major Trauma Triage Study (MATTS), described in detail elsewhere [9].

### Setting

The study was undertaken in four inclusive regional trauma networks: Birmingham, West Yorkshire, North West London, and Severn. These are predominantly served by four separate NHS ambulance services: West Midlands Ambulance Service (WMAS); Yorkshire Ambulance Service (YAS); London Ambulance Service (LAS); and the South-Western Ambulance Service (SWAS) respectively. The study trauma networks represent a diverse range of localities, demographic, socioeconomic and injury profiles. Further details on the participating trauma networks are provided in the supplementary materials.

### Index tests and reference standard

Participating ambulance service triage tools consisted of a checklist of variables applied in parallel. With exception of SWAS, these were grouped into domains. Physiology (‘step 1’) and anatomical injury (‘step 2’) variables are mandatory, indicating bypass and pre-alerting to MTCs when positive. Mechanism of injury (‘step 3’) and special circumstance (‘step 4’) variables are discretionary, prompting consideration of bypass and pre-alerting to MTCs. Ambulance service triage tools are presented in the supplementary materials.

The primary index test under consideration was prehospital provider triage decision making, with a positive result defined as patient transport with a pre-alert call to the MTC. This reflects the dual purposes of a major trauma tirage tool: a) to select patients for bypass to a distant MTC (relevant to patients injured closest to a non-MTC), and b) to inform emergency department pre-alert calls facilitating activation of a hospital trauma team (relevant to patients injured closest to MTC or bypassed). Initial hospital destination, MTC versus non-MTC, was investigated as a secondary index test.

The theoretical diagnostic accuracy of each ambulance service's major trauma triage tool was also examined, representing the simulated application of triage tools. Triage tool variables were assessed according to objective data present in ambulance service records, regardless of the final triage decision or hospital destination, clinical judgement, or hospital destination.

The primary reference standard against which triage decisions were assessed was injured patients who would benefit from expedited MTC care, as characterised by previously published MATTS consensus-based definition [10]. This consists of four domains, comprising: need for critical interventions, presence of significant individual anatomical injuries requiring specialist care, a burden of multiple injuries benefiting from specialist multidisciplinary management, and patient characteristics indicating a capacity to benefit from advanced specialist care. Secondary reference standards were also considered: injury severity score (ISS) ≥16; [11] the need for urgent trauma interventions (the critical interventions domain of the MATTS reference standard); [10] and the MATTS reference standard without open fractures.

### Study population

The source population was all patients presenting with acute injuries to the four participating ambulance services and included trauma networks. The subsequent study population consisted of consecutive patients, irrespective of age, conveyed to a participating trauma network hospital, between 1^st^ November 2019 and 28^th^ February 2020 and meeting study inclusion and exclusion criteria as detailed in Table 1. A random sample of individuals from the study population were included irrespective of reference standard status (‘sub-cohort’). Furthermore, all eligible patients meeting reference standard criteria were identified from the Trauma Audit and Research Network (TARN) database, the English national trauma registry, and included as 'cases' [12]. Data linkage between a) cases meeting reference standard criteria collected by TARN, and b) Emergency medical services (EMS) identified patients with acute injuries, was conducted deterministically where possible based on a unique ambulance service patient report form number shared across both datasets. In cases where exact deterministic matching was not possible due to missing or inaccurate patient report form number, research paramedics performed probabilistic matching by manually reviewing each reference standard positive case in detail. Demographic, non-unique ambulance identifiers (e.g., ambulance call sign) and incident information was used from TARN data to search for a corresponding record in ambulance service databases. All matches were independently confirmed by a second researcher and a match was not confirmed in the presence of any uncertainty or disagreement. The final study sample included patients with complete data available allowing calculation of triage tool diagnostic accuracy.

**Table 1:** Inclusion and exclusion criteria

**Inclusion criteria**	**Exclusion criteria**
Direct conveyance from scene to a hospital in included trauma network	Conveyed to non-participating hospital
Conveyed by participating ambulance service	Conveyed by non-participating ambulance service
Presented during 4 months study period	Not conveyed to hospital
Any age	Non-ambulance presentation
Presented with non-trivial injury to EMS (selected WI code, major trauma pre-alert, trauma-specific intervention)	Death in field
Major trauma triage tool would plausibly be used	Secondary transfer
	Presentation out of study dates
	Trivial injury presentation
	Non-acute injury presentation (>72 hours)
	Medical presentation
	Isolated hypoxic injury (hanging, drowning)
	Isolated burns
	Triage tool exclusions: Traumatic cardiac arrest, Unstable ABC/divert to TU

### Data collection

Patient records for patients sampled in the sub-cohort, or matched to non-sampled reference standard positive cases, were imported into a bespoke research database. Demographic, patient characteristics, physiology, incident, mechanism of injury, interventions, treatments, and clinical assessment information were collected. Relevant electronic closed field data were imported directly where possible, with free text data coded by hand after review of the patients record by research paramedics. Data abstraction was blinded, with all ambulance service data anonymised and reference standard status not available. Data collection was piloted and guided by a pre-specified coding dictionary. Weekly meetings were convened to review data collection, with any uncertainties resolved through consensus. Data was recorded as missing if not present in closed fields, or if not possible to infer from free text fields. Following data collection, range and consistency checks were performed with implausible values set to missing.

TARN data collection has been reported in detail previously [12, 13]. Each submitted TARN case from participating hospitals during the study period was coded centrally by TARN data analysts according to primary and secondary reference standard criteria. Anonymised data for reference standard positive cases were then imported into a study database for review by research paramedics. Eligibility against inclusion and exclusion criteria was confirmed manually.

### Statistical analyses

The analysis proceeded in six stages. Firstly, the derivation of study population, parent cohort, sub-cohort, and reference standard positive cases were enumerated and delineated graphically using flow charts. Secondly, the study sample was characterised, with patient demographics, injury features and missing data examined using descriptive statistics. Thirdly, in the main analysis, the diagnostic accuracy of prehospital provider triage decision for all patients was investigated against the primary reference standard. Sensitivity and specificity were calculated with their 95% confidence intervals, overall and stratified by each ambulance service. Fourthly, the accuracy of triage decisions across different age groups was evaluated for the whole sample, with sensitivity and specificity calculated for children (aged <16 years) and subgroups defined by adult age deciles. Finally, in secondary analyses, the theoretical accuracy of each ambulance service triage tool was assessed. Triage tools were coded algorithmically according to their stated variable thresholds against the observed data. The first recorded vitals sign was used for physiology variables. Where triage tool variables were defined as sustained physiology values, two or more consecutive values meeting the threshold were required. Two independent statisticians undertook coding to ensure accuracy. Cumulative sensitivity and specificity of sequential triage tool steps, including discretionary steps, based on recorded data were calculated in the full study sample. Results were displayed graphically using plots of paired sensitivity/specificity and compared to the accuracy of provider triage decisions. In sensitivity analyses the main analysis was repeated for alternative index tests (MTC Vs non-MTC initial destination) and reference standards (injury severity score ≥16, the need for urgent trauma interventions, and the MATTS open reference standard without open fractures).

Analyses were conducted in R Statistical Software (v4.3.0; R Core Team 2023) and STATA version 17.0 (StataCorp. 2016. Stata Statistical Software: Release 17. College Station, TX: StataCorp LP). Unweighted summary statistics were reported separately for the case-cohort sample characteristics, diagnostic accuracy metrics, and reference standard prevalence. The unit of analysis was the individual incident. Direct patient identifiers were unavailable, and it was therefore not possible to account for clustering from recurrent incidents in the same patient.

### Funding, ethical approval and data governance

This study was undertaken as part of the Major Trauma Triage Study (MATTS) project, aiming to develop a new national triage tool, funded by the National Institute of Health Research Health Technology Agency Assessment Programme (NIHR HTA ref: 17/16/04) [9]. Ethical approval was provided by Yorkshire and The Humber - Bradford Leeds Research Ethics Committee (Reference: 19/YH/0197). A study protocol and statistical analysis plan were pre-specified.

## Results

### Sample derivation and characteristics

**Fig. 1:**
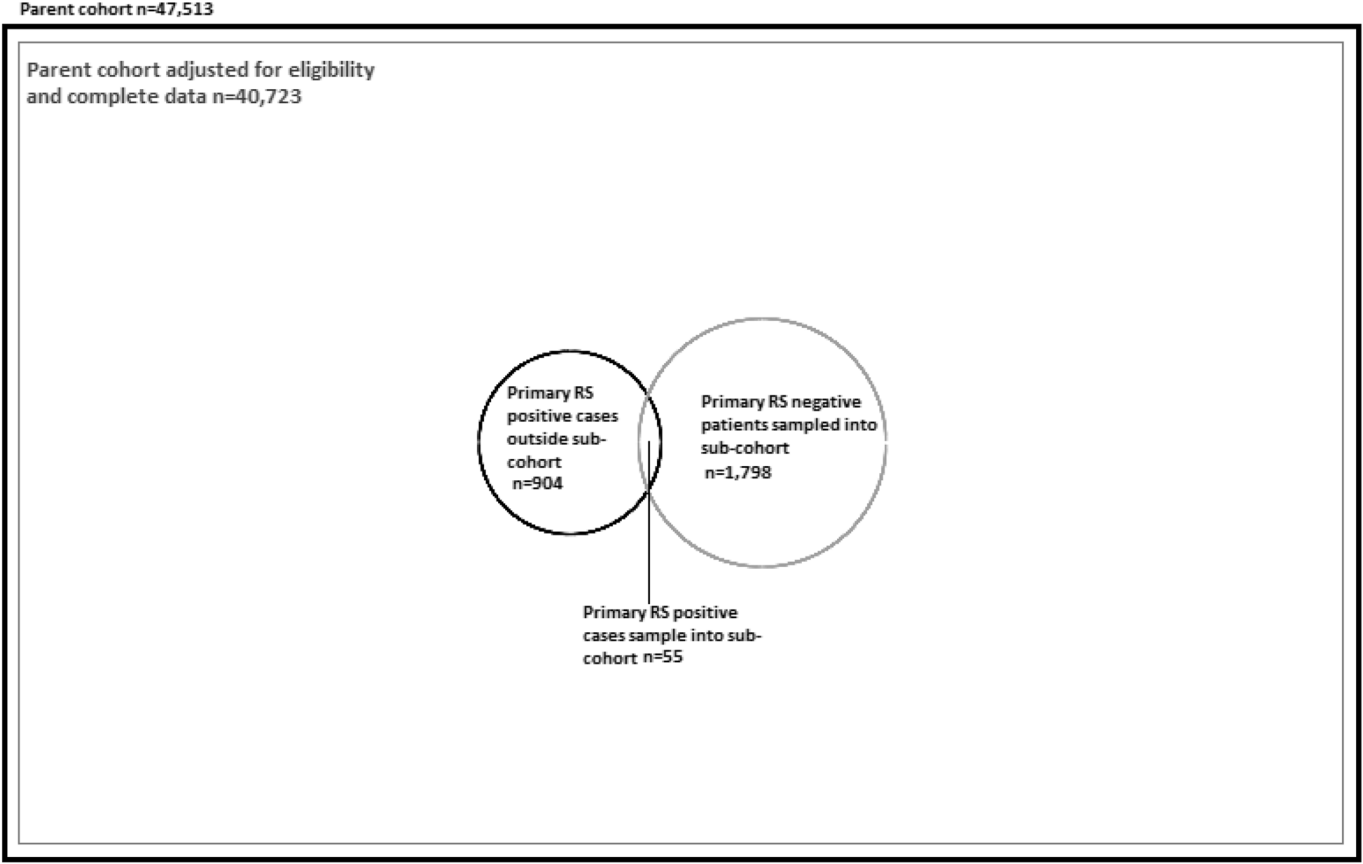
Case-cohort derivation of primary reference standard positive and negative cases in sub-cohort and parent cohort. Area of circles and squares is proportional to the number of patients

**Fig. 2:**
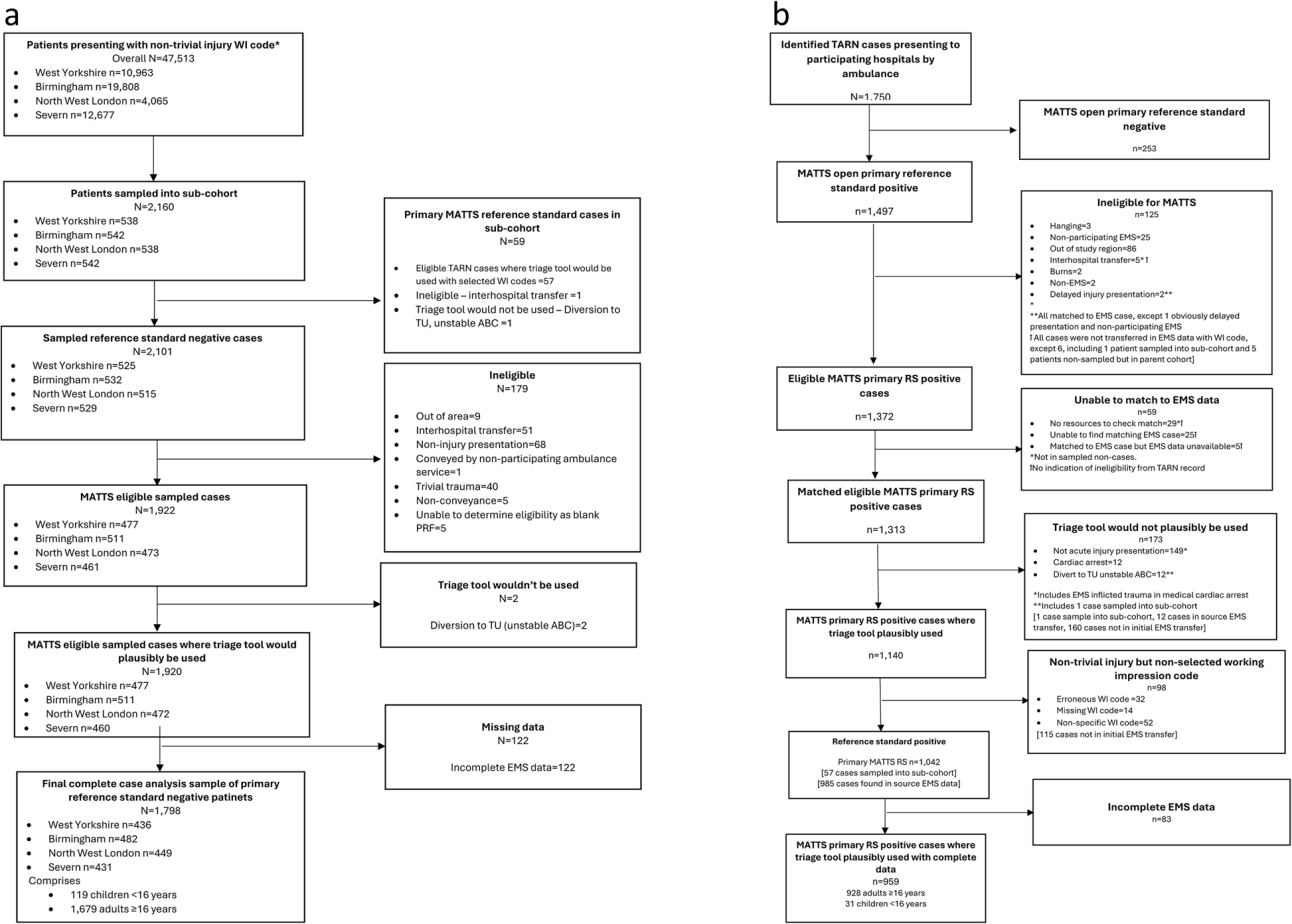
Derivation of primary reference standard positive and negative patients

Between 1st November 2019 and 28th February 2020, 47,513 patients with non-trivial injury working impression codes were conveyed to included trauma network hospitals by participating ambulance services, forming the source population. Overall, 1,853 eligible patients with complete data were sampled into the sub-cohort, comprising 55 patients (54 adults, 1 child) meeting primary reference standard criteria, and 1,798 patients (1,679 adults, 119 children) who were primary reference standard negative. During the same study period 959 TARN cases (928 adults, 31 children) met inclusion criteria, were successfully matched to a corresponding ambulance service record, and had complete data. Derivation of the parent cohort, sub-cohort, and study samples for primary reference standard positive and negative patients are detailed in Figs. 1 and 2.

The overall prevalence of major trauma meeting the primary reference standard definition in eligible sub-cohort patients with complete data was 3.1% (*n*=54/1,798, 95% CI 2.3 – 4.0). Included patients presenting to ambulance services with non-trivial injury were predominantly elderly (median age 73 years), female (52.6%), and sustained accidental (88.1%) blunt trauma (97.7%) from ground level falls (70.0% of injury mechanisms). Characteristics of the included complete case study sample are detailed in Table 2.

**Table 2:** Characteristics of complete case case-cohort study sample

**Variable**	**Category**	**Reference standard positive (*n*=/959)**	**Reference standard positive summary statistic**	**Reference standard negative (*n*=/1,798)**	**Reference standard negative summary**
**Demographics**					
Age (years)	Age	959	Median 61	1,798	Median 73
			IQR 38-79		IQR 40-84
			Range 0-102		Range 0-103
Age groups (%)	Child <16	31	3.2%	119	6.6%
	Adult 16-64.9	491	51.2%	644	35.8%
	Elderly >65	437	45.6%	1035	57.6%
Sex (%)	Female	398	41.5%	944	53.2%
	Male	560	58.5%	831	46.8%
**Injury characteristics**					
Mode of injury	Blunt	902	94.1%	1,758	97.8%
	Penetrating	57	5.9%	40	2.2%
Mechanism of injury	Cutting/piercing/stabbing	56	5.8%	40	2.2%
	Gunshot	1	0.1%	0	0%
	Fall>1m	232	24.2%	109	6.1%
	Fall<1m	339	35.4%	1,274	70.9%
	RTA (motorcycle)	40	4.2%	20	1.1%
	RTA (motor vehicle)	76	7.9%	66	3.7%
	RTA (bicycle)	30	3.1%	22	1.2%
	RTA (pedestrian)	107	11.2%	45	2.5%
	Struck by/collision with object	45	4.7%	85	4.7%
	Struck by/collision with person	21	2.2%	106	5.9%
	Other / unknown	12	1.3%	31	1.7%
**Vital signs**					
SBP	Median	959	137	1,798	142
	IQR		120-156		126-161
	Range		41-279		50-258
RR	Median	959	20	1,798	18
	IQR		17-22		16-20
	Range		0-56		11-67
Peripheral oxygen saturations	Median	959	97	1,798	97
	IQR		94-99		96-99
	Range		0-100		68-100
GCS	Median	959	15	1,798	15
	IQR		14-15		15-15
	Range		3-15		3-15
**Incident characteristics**					
HEMS response	Yes	185	19.3%	23	1.3%
	No	774	80.7%	1775	98.7%
Mode of transport	Land	936	97.6%	1,795	99.8%
	Helicopter	23	2.4%	3	0.2%
High priority leaving scene	Yes	544	56.7%	163	9.0%
	No	415	43.3%	1635	91.0%
Closest hospital	LEH	66	6.9%	191	10.6%
	TU	702	73.2%	1,301	72.4%
	MTC	190	19.8%	304	16.9%
	Unknown/unclear	1	0.1%	2	0.1%
Injury severity					
Injury Severity Score	Injury Severity Score	959	Median 18		
			IQR 13-25		
			Range 4-66		
AIS body region injured	Head	508	53.0%		
	Face	140	14.6%		
	Thorax	385	40.2%		
	Abdomen	198	20.6%		
	Extremities	483	50.4%		
	External	316	33.0%		
Any urgent intervention	No	664	69.2%		
	Yes	295	30.8%		
Critical care admission	No	850	88.6%		
	Yes	109	11.4%		
Died within 30 days	No	880	91.8%		
	Yes	79	8.2%		

*GCS* Glasgow Coma Scale, *ISS* Injury severity score, *RR* Respiratory rate, *SBP* Systolic blood pressure

### Accuracy of prehospital provider triage decisions

**Fig. 3:**
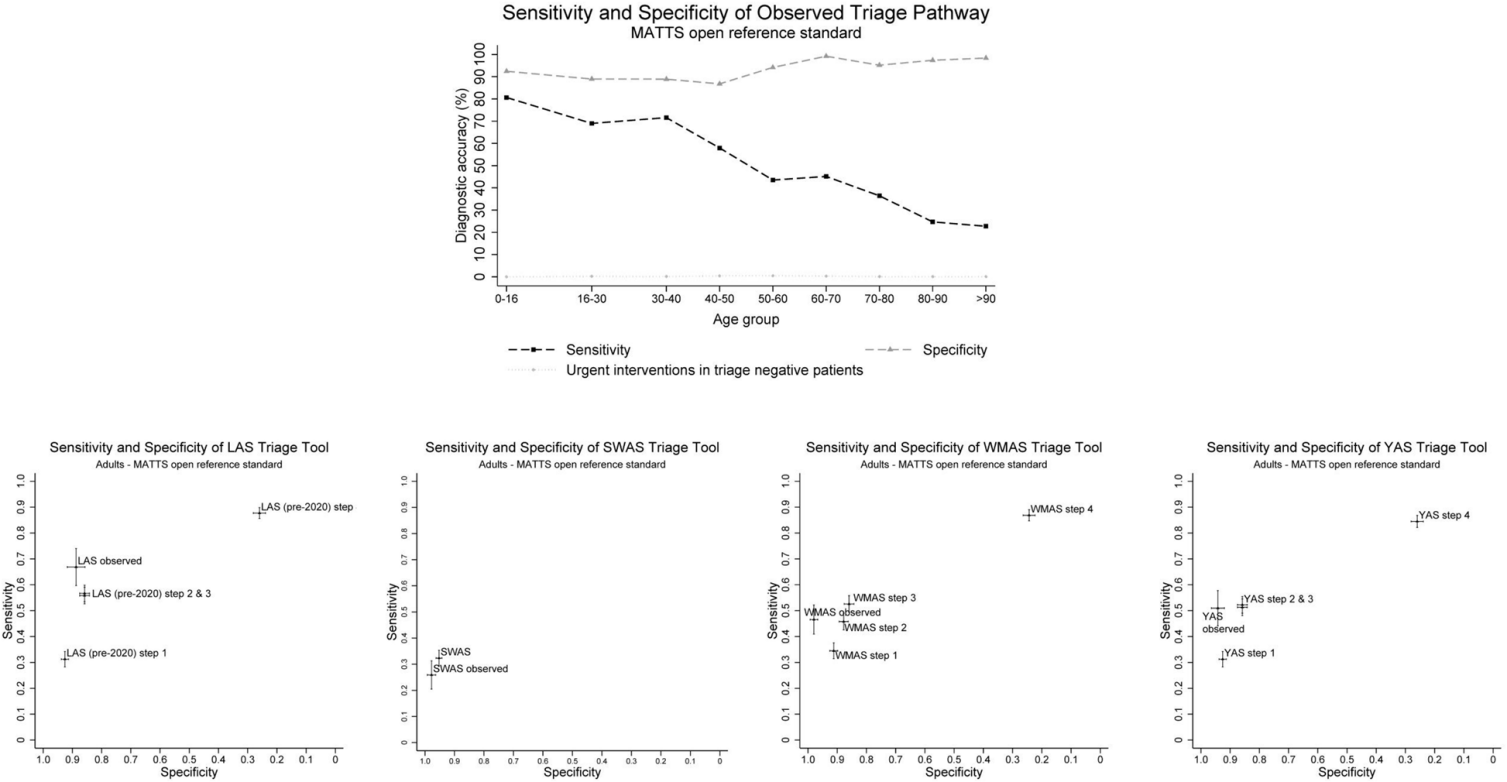
Top panel: Sensitivity and specificity of observed triage decisions across different patient age groups; Bottom panel: Receiver operating characteristic curves for participating ambulance services observed triage decisions and theoretical triage tool results evaluated against the primary MATTS reference standard in patients aged over 16 years

Observed prehospital provider triage decisions (i.e., whether the patient was pre-alerted to the MTC, regardless of the indicated triage tool result) demonstrated overall pooled sensitivity of 46.7% (95% CI 43.5-49.9) and specificity of 94.5% (95% CI 93.4-95.6) for the MATTS reference standard. However, there was a marked variation in triage decisions across ambulance services, ranging from low sensitivity (26%) and high specificity (98%) in SWAS, to higher sensitivity (67%) and lower specificity (89%) in LAS, as shown in Table 3 and Fig. 3. There was a clear trend of decreasing sensitivity and increasing specificity from younger to older ages. Sensitivity was 80.6% in under 16 years falling to 22.7% in over 90 years. Corresponding specificity was 92.4% in under 16 years, increasing to 98.4% in patients over 90 years (Fig. 3). Of note, a small proportion (<1% across all age groups) of patients not conveyed to a MTC with a pre-alert underwent an urgent trauma intervention.

**Table 3:** Diagnostic accuracy metrics for ambulance service triage tools and prehospital provider triage decisions (conveyed to MTC with pre-alert) evaluated against the primary MATTS reference standard

**Tool number**	**Tool name**	**n**	**TP**	**FP**	**TN**	**FN**	**Sensitivity**	**Sens LCL**	**Sens UCL**	**Specificity**	**Spec LCL**	**Spec UCL**	**Positive LR**	**Positive LR LCL**	**Positive LR UCL**	**Negative LR**	**Negative LR LCL**	**Negative LR UCL**
1	LAS Step 1	2,757	303	151	1,647	656	0.32	0.29	0.35	0.92	0.90	0.93	3.76	3.15	4.50	0.75	0.64	0.88
2	LAS Steps 1/2	2,757	540	266	1,532	419	0.56	0.53	0.59	0.85	0.84	0.87	3.81	3.36	4.31	0.51	0.45	0.59
3	LAS Steps 1/2/3	2,757	548	267	1,531	411	0.57	0.54	0.60	0.85	0.84	0.87	3.85	3.40	4.35	0.50	0.44	0.57
4	LAS Steps 1/2/3/4	2,757	836	1,272	526	123	0.87	0.85	0.89	0.29	0.27	0.31	1.23	1.19	1.28	0.44	0.37	0.52
5	SWAS	2,757	319	135	1,663	640	0.33	0.30	0.36	0.92	0.91	0.94	4.43	3.68	5.33	0.72	0.61	0.85
6	WMAS Step 1	2,757	333	164	1,634	626	0.35	0.32	0.38	0.91	0.90	0.92	3.81	3.21	4.51	0.72	0.62	0.84
7	WMAS Steps 1/2	2,757	443	224	1,574	516	0.46	0.43	0.49	0.88	0.86	0.89	3.71	3.22	4.27	0.61	0.54	0.70
8	WMAS Steps 1/2/3	2,757	509	261	1,537	450	0.53	0.50	0.56	0.85	0.84	0.87	3.66	3.22	4.15	0.55	0.48	0.63
9	WMAS Steps 1/2/3/4	2,757	827	1,295	503	132	0.86	0.84	0.88	0.28	0.26	0.30	1.20	1.15	1.24	0.49	0.42	0.58
10	YAS Step 1	2,757	308	168	1,630	651	0.32	0.29	0.35	0.91	0.89	0.92	3.44	2.90	4.08	0.75	0.64	0.87
11	YAS Steps 1/2	2,757	501	283	1,515	458	0.52	0.49	0.55	0.84	0.83	0.86	3.32	2.94	3.75	0.57	0.50	0.64
12	YAS Steps 1/2/3	2,757	510	284	1,514	449	0.53	0.50	0.56	0.84	0.83	0.86	3.37	2.98	3.80	0.56	0.49	0.63
13	YAS Steps 1/2/3/4	2,757	809	1,289	509	150	0.84	0.82	0.87	0.28	0.26	0.30	1.18	1.13	1.22	0.55	0.48	0.64
14	LAS triage decisions	622	118	50	399	55	0.68	0.61	0.75	0.89	0.86	0.92	6.13	4.63	8.11	0.36	0.25	0.50
15	SWAS triage decisions	687	66	9	422	190	0.26	0.20	0.31	0.98	0.97	0.99	12.35	6.26	24.35	0.76	0.40	1.45
16	WMAS triage decisions	801	154	11	471	165	0.48	0.43	0.54	0.98	0.96	0.99	21.15	11.67	38.36	0.53	0.29	0.96
17	YAS triage decisions	647	108	25	411	103	0.51	0.44	0.58	0.94	0.92	0.96	8.93	5.97	13.35	0.52	0.35	0.78
18	Overall triage decisions	2,757	446	95	1,703	513	0.47	0.43	0.50	0.95	0.94	0.96	8.80	7.16	10.83	0.56	0.46	0.69

*TP* True positive, *FP* False positive, *TN* True negative, *FN* False negative, *LCL* Lower confidence interval limit, *UCL* Upper confidence interval limit, *LR* Likelihood ratio

### Theoretical accuracy of ambulance service triage tools

There was variation in sensitivity and specificity across ambulance service triage tools following theoretical application of mandatory steps: SWAS (0.32/0.95), WMAS (0.46/0.88), YAS (0.51/0.86), LAS (0.56/0.86). Where relevant, theoretical application of discretionary mechanism of injury (step 3) variables appeared to provide a small increase in sensitivity, with a compensatory small reduction in specificity. Adding special circumstances (step 4) variables resulted in much higher sensitivity, but a very large fall in specificity (Fig. 3). Prehospital provider triage decisions differed from the indicated triage tool result across all ambulance services, with ambulance service clinician judgement resulting in higher specificity than mandatory triage tool steps (Table 3, Fig. 3).

### Sensitivity analyses

Results were largely unchanged in sensitivity analyses examining the secondary ISS≥16 reference standard and omitting open fractures from the primary reference standard. A large increase in sensitivity was seen for both the theoretical application of triage tools, and actual triage decisions (80.0%), when evaluated against the urgent trauma interventions reference standard. Specificity was not materially changed and remained high (94.0%). Sensitivity was slightly increased (57.5%), and specificity decreased (78.6%) for the primary reference standard when prehospital triage decisions were evaluated according to initial hospital destination (MTC Vs non-MTC), regardless of whether a pre-alert was provided. Full details are provided in the supplementary materials.

## Discussion

### Summary

Patients presenting to four English trauma networks by ambulance with non-trivial injury were most commonly elderly females with blunt trauma from ground level falls. The overall prevalence of major trauma was low (3.1%). Observed prehospital provider triage decisions demonstrated overall pooled sensitivity of 46.7% (95% CI 43.5-49.9) and specificity of 94.5% (95% CI 93.4-95.6) for the primary reference standard. However, accuracy was heterogenous with SWAS demonstrating lower sensitivity and higher specificity than other ambulance services. There was a clear trend of decreasing sensitivity and increasing specificity from younger to older ages. Prehospital provider triage decisions did not always follow theoretical triage tool results, with ambulance service triage decisions demonstrating higher specificity. Sensitivity of triage decisions was increased for urgent trauma interventions (80%, 95%CI 75.0-84.0), with specificity remaining high (94%, 95%CI 93.0-95.0).

### Interpretation

Statistical measures of diagnostic accuracy are typically reported in terms of sensitivity and specificity [14]. These metrics may be counterintuitive as they are defined retrospectively by disease status, rather than the clinically relevant probability of a condition being present when the test result in known [15]. There is evidence that graphical representations, predictive values, natural frequencies, or using likelihood ratios, promotes better understanding [15–17]. A range of visual interpretations of the main study findings are therefore presented in the supplementary materials. Taking a Bayesian system-level approach, on average there is a 3.1% chance of major trauma when an acutely injured patient presents to ambulance services. After a positive triage decision for bypassing/pre-alerting the MTC there will be a 22% chance that patient will turn out to have major trauma. Conversely, if the triage decision is negative then there will be a 98% chance that the patient does not have major trauma [18].

For triage tools structured as a 'checklist', diagnostic accuracy could be manipulated by changing the number, type, and thresholds of included variables. This results in an inevitable trade-off between sensitivity and specificity, where the number of false positive cases increases as false negatives are reduced. Sensitivity is often prioritised, for example the ACS-COT has published a target of >95% for field triage, with a consequent penalty of reduced specificity (ASC-COT targets 65-70%) [19]. However, in low prevalence settings this approach would lead to poor positive predictive values and many false positives [20]. Economic modelling has confirmed that prioritising sensitivity is not cost-effective, and specificity should be favoured [21–24]. It is interesting that real-life prehospital triage decisions are closely aligned to these empirical cost-effectiveness targets.

The index test definition for positive prehospital triage is open to debate. Our primary definition of a pre-alert call to the MTC, reflects the dual purposes of a major trauma triage tool of selecting patients for bypass to a distant MTC and facilitating emergency department pre-alert calls. It could be argued that transport to a MTC is the most important factor. However, this would not account for delayed resuscitation and treatment in cases not pre-alerted to the ED. It could also be misleadingly influenced by the proportion of patients injured within the MTC catchment area, as these patients' destination is fixed. Alternatively, from a system perspective, the final common pathway for major trauma patients' is reception into a MTC resuscitation area with hospital trauma team activation. However, full hospital trauma team assessment may not be required in stable patients, activation criteria may differ from triage tool variables, and deployment is outside the control of prehospital providers.

The reference standard against which triage decisions should be assessed is also arguable. The traditionally used Injury Severity Score has many limitations, not least its focus on the degree of anatomical trauma *per se*, rather than the potential to benefit from MTC care [11]. Intervention based reference standards, such as the US consensus definition, may better reflect the need for MTC care, but do not account for benefit arising from MTC supportive care and rehabilitation [10, 25]. Operationalisation in triage research is also potentially challenged by the absence of counterfactual information, and cases may be incorrectly classified as false negatives if they did not receive an intervention that was indicated due to lack of availability or expertise outside the MTC. Evaluation of multiple reference standards will provide a comprehensive and holistic assessment of triage performance.

Differences were apparent in observed prehospital triage, and calculated theoretical triage tool accuracy, across participating trauma networks. The overall pooled accuracy results should therefore be interpreted judiciously. The heterogeneity of results may reflect the use of different ambulance service triage tools. Theoretical accuracy of mandatory triage tool steps (SWAS most specific, LAS most sensitive, WMAS/YAS intermediate), appeared to correlate with real-life triage decision making (SWAS most specific, LAS most sensitive, WMAS/YAS intermediate). Alternatively, the variation could reflect differences in philosophies (inclusive versus exclusive focus), geographies (more urban Vs more rural), or network organisation (e.g., extent of remote decision support available from trauma desks).

Prehospital providers triage decisions were observed to differ from those indicated by triage tools, apparently improving triage performance. This could result from discretionary triage tool use in selected patients, application of additional clinical judgement to over-rule indicated triage tool results, or the influence of applying discretionary triage tool steps. Previous qualitative research has demonstrated that prehospital triage making is often heuristic with triage tools used less commonly as experience increases [26]. It is perhaps not surprising that subjective real-life decisions outperformed objective triage tool accuracy, as clinical judgement has been consistently demonstrated to be superior in multiple studies across many disease areas [27, 28]. This has important implications, as to benefit from better overall triage performance achieved through subjective clinical judgement, some incorrect individual clinical decisions will need to be accepted; and clinical governance strategies to increase triage tool use and adherence may be counterproductive. Spectrum effects were apparent across different age groups with decreasing sensitivity and increasing specificity for triage decisions from younger to older ages for the primary reference standard. Interestingly, the proportion of false negatives requiring urgent trauma interventions remained negligible (<1%), perhaps reflecting provider judgement in selecting patients perceived to benefit most from MTC care.

The four included trauma networks are representative of the broader English population, including a diverse mix of urbanisation, socioeconomic status, geographies, and injury profiles. The results of this study should therefore be generalisable throughout the UK National Health Service. In response to the COVID-19 pandemic, new triage tools have been implemented in LAS and SWAS, which may influence contemporary triage. External validity to other settings is limited. Different injury patterns (e.g., higher numbers of gun-shot wounds), alternative health system models (state Vs insurance Vs private funding), trauma network organisation (level of training, inclusive Vs exclusive), medicolegal risk, and patient demographics (e.g., population age profiles) in other settings could strongly influence triage decision making.

### Comparison to literature

Two recent systematic reviews have examined real-life major trauma triage decisions and compliance with theoretical triage tool results. Van Rein (2018a) evaluated prehospital triage decisions, defined by initial hospital destination, in 33 studies [29]. The findings were limited by poor methodological quality and very heterogenous results were reported with sensitivity ranging from 32% to 99% and specificity from 1% to 99%. Notwithstanding the different populations and trauma system organisation seen in the predominantly US settings, results from the better-quality studies were not disimilar to the current findings. It was also concluded, in common with the current study, that EMS provider judgment added value to triage protocols in the identification of severely injured patients. Van Rein (2018b) also investigated compliance, reviewing 11 studies comparing objective triage tool results with actual triage destinations [30]. The methodological quality of most studies was again poor, with widely disparate compliance rates between 21% and 91% reported. One study with good methodological quality showed, in common with our findings, that the triage protocol identified only a minority of severely injured patients, and a tendency to transport elderly trauma patients to lower-level trauma centres, even if the patient met one or more triage criteria.

### Limitations

This study has several strengths. The risk of information bias was minimised by following recommendations for collection of routine data in retrospective studies [31, 32]. Other common sources of systematic error in diagnostic accuracy studies were avoided, including a 'two-gate' case-control study design; and test, diagnostic review, partial verification, incomplete verification, incorporation, and disease progression biases [33]. However, there are some potential limitations. The use of routine data may have resulted in index test or reference standard misclassification. Selection bias could arise from imperfect matching of ambulance service and TARN data and complete case analyses, omitting cases with missing data. However, the matching rate was high (>95%) and missing data rate was low (<10%). Furthermore, reference standard classification is dependent on encompassing TARN inclusion criteria, complete case ascertainment by TARN, and accurate matching of TARN and prehospital data.

## Conclusions

Prehospital decision making for injured patients in English trauma networks demonstrated high specificity and low sensitivity, consistent with the targets for cost-effective triage defined in previous economic evaluations. Actual triage decisions differed from theoretical triage tool results, with a clear trend of decreasing sensitivity and increasing specificity from younger to older ages. Further research could usefully explore factors associated with triage tool compliance, such as included variables, mechanism of injury, injury timing and location, and patient characteristics.

### Supplementary Information

Supplementary Material 1. 

Supplementary Material 2. 

Supplementary Material 3. 

## Data Availability

The anonymised data and full reproducible analysis code is available on reasonable request, subject to ethical approvals and appropriate data sharing agreements.

## Abbreviations

MTC: Major Trauma Centre
MATTS: Major Trauma Triage Study
WMAS: West Midlands Ambulance Service
YAS: Yorkshire Ambulance Service
LAS: London Ambulance Service
SWAS: South-Western Ambulance Service
EMS: Emergency medical services
TARN: Trauma Audit and Research Network
ISS: Injury severity score
