# Supplementary material for: The accuracy of prehospital triage decisions in English trauma networks – a case-cohort study
Source: Scand J Trauma Resusc Emerg Med. 2024 May 21;32:47. doi: 10.1186/s13049-024-01219-9 (PMC11110388; doi:10.1186/s13049-024-01219-9)
Supplement: Supplementary file 1 — Supplementary Material 1. [file 13049_2024_1219_MOESM1_ESM.docx]

**How is care for major trauma patients currently organised in England?**

The care of seriously injured patients in England was noted to be sub-optimal in a 2007 report by the National Confidential Enquiry into Patient Outcome and Death, where 60% of cases demonstrated deficiencies in organisational or clinical aspects of care.^1^ A subsequent 2010 National Audit Office enquiry confirmed these findings, highlighting the ad hoc organisation of trauma care, with unacceptable variations in mortality rates, depending on where and when a patient received treatment.^2^

In response, trauma care in England was reconfigured with the introduction of inclusive regional trauma networks in 2011, based on the established model of care for injured patients in North America.^3^ Such networks aim to ensure transport of patients injured within a defined geographical area to a hospital matched to their clinical need. These systems of care consist of a central major trauma centre (MTC) hospital providing specialist resuscitation, definitive care, and rehabilitation to the highest acuity and most seriously injured patients. Non-specialist general hospitals, termed trauma units, manage lower acuity and less seriously injured patients, and can provide stabilisation and transfer of the more seriously injured to MTCs when needed. Other acute hospitals, which would not routinely manage significantly injured patients, are designated Local Emergency Hospitals. Management, training, and governance structures are incorporated to coordinate patient management and ensure high quality care is delivered. Trauma networks have subsequently been similarly introduced in Northern Ireland (2017), Scotland (2018) and Wales (2021).

In accordance with NICE major trauma service delivery guidelines, NHS ambulance services therefore use pre-hospital triage tools within regional trauma networks.^4^ Their primary purpose is to identify which patients injured within the catchment areas of TUs and LEHs might benefit from prolonged transportation to distant MTCs, bypassing the closer non-specialist hospital. Furthermore, relevant to patients injured in both MTC and non-MTC catchment areas, triage tools have an important secondary role to inform emergency department (ED) pre-alert calls, facilitating patient reception into critical care areas and activation of multi-disciplinary hospital trauma teams to provide rapid, specialised, and coordinated assessment and resuscitation.

Prehospital care is routinely provided ambulance service technicians (entry level prehospital providers) or paramedics (senior prehospital providers), with assistance provided by emergency care assistants (ambulance drivers with basic clinical training). Multiple enhanced care resources may be available to respond to suspected major trauma cases, including, helicopter emergency medical service (HEMS), pre-hospital physician-paramedic teams, or specialist critical care paramedics.^5, 6^ Major trauma specialist services may also be spread across different hospitals, within or across NHS trusts and local healthcare organisations. Moreover, hospital TU and MTC designations may differ depending on paediatric or adult presentations. Ambulance services operate 'trauma desks', coordinate the care of patients in the field (including enhanced care teams), providing remote telephonic clinical guidance on management of injured patients, destination advice, and liaison with hospital emergency departments.


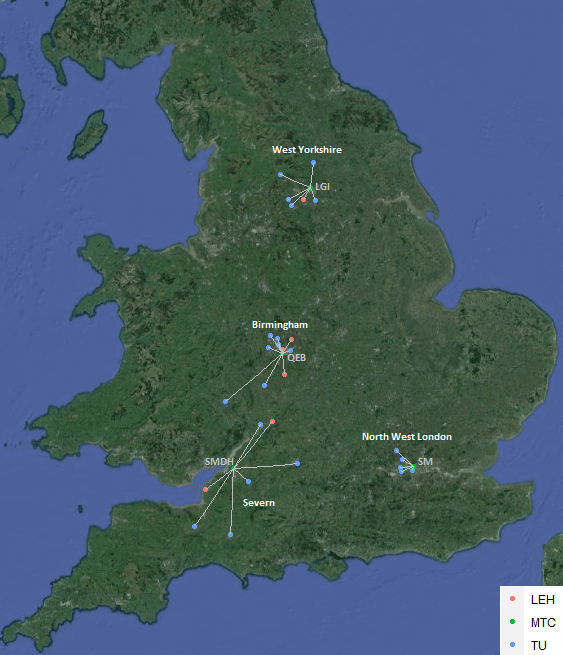


**Participating trauma networks**

LGI: Leeds General Infirmary, QEB: Queen Elizabeth’s Hospital Birmingham, SMDH: Southmead Hospital, SM: ST Mary’s Hospital. Note, Bristol Royal Infirmary (TU) and Bristol Children’s Hospital (MTC) are obscured by Southmead Hospital. Similarly, St James Hospital (TU) is obscured by Leeds General Infirmary.

**Included trauma networks and hospitals**

| **Trust** | **Hospital** | **Designation-Adults** | **Designation-Children** |
| --- | --- | --- | --- |
| **Severn Network** | | | |
| - Mixed urban / rural geography - Large city (Bristol) - Surrounding rural, semi-rural areas and smaller towns | | | |
| North Bristol NHS Trust | Southmead Hospital | MTC | N/A |
| University Hospitals Bristol NHS Foundation Trust | Bristol Royal Hospital for Children | N/A | MTC |
| University Hospitals Bristol NHS Foundation Trust | Bristol Royal Infirmary | TU | N/A |
| Royal United Hospitals Bath NHS Foundation Trust | Royal United Hospital Bath | TU | TU |
| Somerset NHS Foundation Trust | Yeovil Hospital | TU | TU |
| Somerset NHS Foundation Trust | Musgrove Park Hospital | TU | TU |
| Great Western Hospitals NHS Foundation Trust | Great Western Hospital (Swindon) | TU | TU |
| Gloucestershire Hospitals NHS Foundation Trust | Gloucestershire Royal Hospital | TU | TU |
| University Hospitals Bristol NHS Foundation Trust | Western General Hospital | LEH | LEH |
| Gloucestershire Hospitals NHS Foundation Trust | Cheltenham General Hospital | LEH | LEH |
| **North West London Network** | | | |
| - Mixed predominantly urban geography (North West London) | | | |
| Imperial College Healthcare NHS Trust | St Mary's Hospital | MTC | MTC |
| Hillingdon Hospitals NHS Foundation Trust | Hillingdon Hospital | TU | TU |
| Chelsea and Westminster Hospital NHS Foundation Trust | Chelsea and Westminster Hospital | TU | TU |
| Chelsea and Westminster Hospital NHS Foundation Trust | West Middlesex University Hospital | TU | TU |
| London North West University Healthcare NHS Trust | Ealing Hospital | TU | TU |
| London North West University Healthcare NHS Trust | Northwick Park Hospital | TU | TU |
| West Hertfordshire Teaching Hospitals NHS Trust | Watford General Hospital | TU | TU |
| **West Yorkshire Network** | | | |
| - Predominantly urban and semi-rural geography - Large cities (Leeds and Bradford) - Surrounding semi-rural areas and towns | | | |
| Leeds Teaching Hospitals NHS Trust | Leeds General Infirmary | MTC | MTC |
| Leeds Teaching Hospitals NHS Trust | St James University Hospital | TU | TU |
| Airedale NHS Foundation Trust | Airedale General Hospital | TU | TU |
| Bradford Teaching Hospitals NHS Foundation Trust | Bradford Royal Infirmary | TU | TU |
| Calderdale and Huddersfield NHS Foundation Trust | Calderdale Royal Hospital | TU | TU |
| Mid Yorkshire Hospitals NHS Trust | Dewsbury and District Hospital | LEH | LEH |
| Harrogate and District NHS Foundation Trust | Harrogate District Hospital | TU | TU |
| Calderdale and Huddersfield NHS Foundation Trust | Huddersfield Royal Infirmary | TU | TU |
| Mid Yorkshire Hospitals NHS Trust | Pinderfields General Hospital | TU | TU |
| **Birmingham Network** | | | |
| - Mixed urban / rural geography - Large city (Birmingham connurbation) - Surrounding rural, semi-rural areas and smaller towns | | | |
| University Hospitals Birmingham NHS Foundation Trust | Queen Elizabeth Birmingham | MTC | N/A |
| Birmingham Women’s and Children’s NHS Foundation Trust | Birmingham Children’s Hospital | N/A | MTC |
| University Hospitals Birmingham NHS Foundation Trust | Heartlands Hospital | TU | TU |
| Sandwell and West Birmingham Hospitals NHS Trust | Birmingham City Hospital | LEH | LEH |
| University Hospitals Birmingham NHS Foundation Trust | Good Hope Hospital | LEH | LEH |
| Wye Valley NHS Trust | Hereford General Hospital | TU | TU |
| Royal Wolverhampton NHS Trust. | New Cross Hospital | TU | TU |
| The Dudley Group NHS Foundation Trust | Russells Hall Hospital | TU | TU |
| Sandwell and West Birmingham Hospitals NHS Trust | Sandwell District General Hospital | TU | TU |
| Walsall Healthcare NHS Trust | Walsall Manor Hospital | TU | TU |
| Worcestershire Acute Hospitals NHS Trust | Worcestershire Royal Hospital | TU | TU |
| Worcestershire Acute Hospitals NHS Trust | Alexandria Hospital | LEH | LEH |

**REFERENCES**

1. Death NCEiPOa. *NECPOD Report. Trauma Who Cares?*; 2007.

2. Office NA. *Major Trauma*. London, UK; 2010.

3. McCullough AL, Haycock JC, Forward DP, Moran CG. II. Major trauma networks in England. *BJA: British Journal of Anaesthesia* 2014;**113**:202-6. <https://doi.org/10.1093/bja/aeu204>

4. National Clinical Guideline C. Major Trauma: Service Delivery. 2016.

5. Hoyt DB, Coimbra R. Trauma systems. *Surg Clin North Am* 2007;**87**:21-35, v-vi. <https://doi.org/10.1016/j.suc.2006.09.012>

6. Yates D. Regional trauma systems. *BMJ* 1997;**315**:1321-2.
