# Supplementary material for: The accuracy of prehospital triage decisions in English trauma networks – a case-cohort study
Source: Scand J Trauma Resusc Emerg Med. 2024 May 21;32:47. doi: 10.1186/s13049-024-01219-9 (PMC11110388; doi:10.1186/s13049-024-01219-9)
Supplement: Supplementary file 2 — Supplementary Material 2. [file 13049_2024_1219_MOESM2_ESM.pptx]

## Slide 1
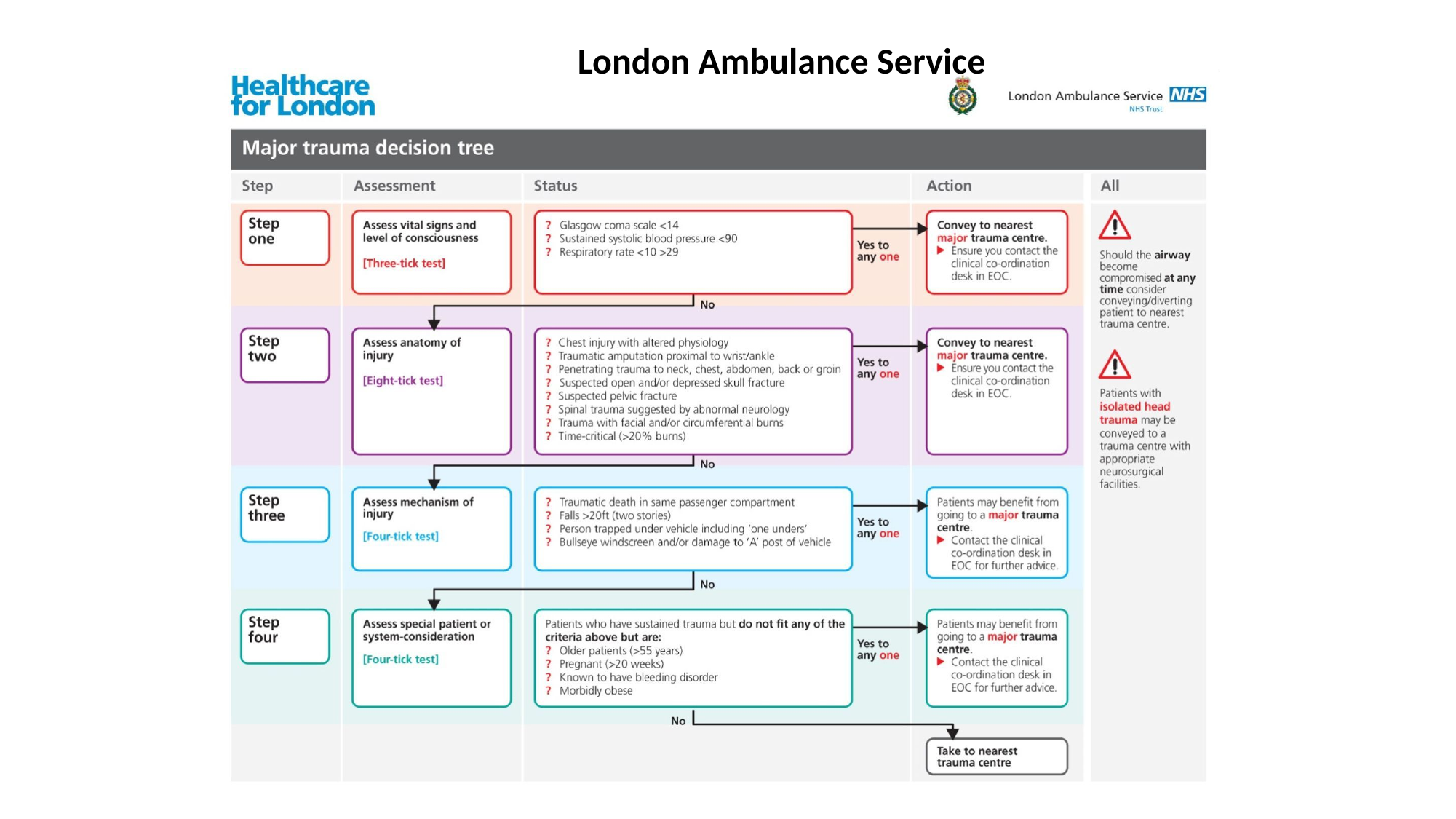

London Ambulance Service

## Slide 2
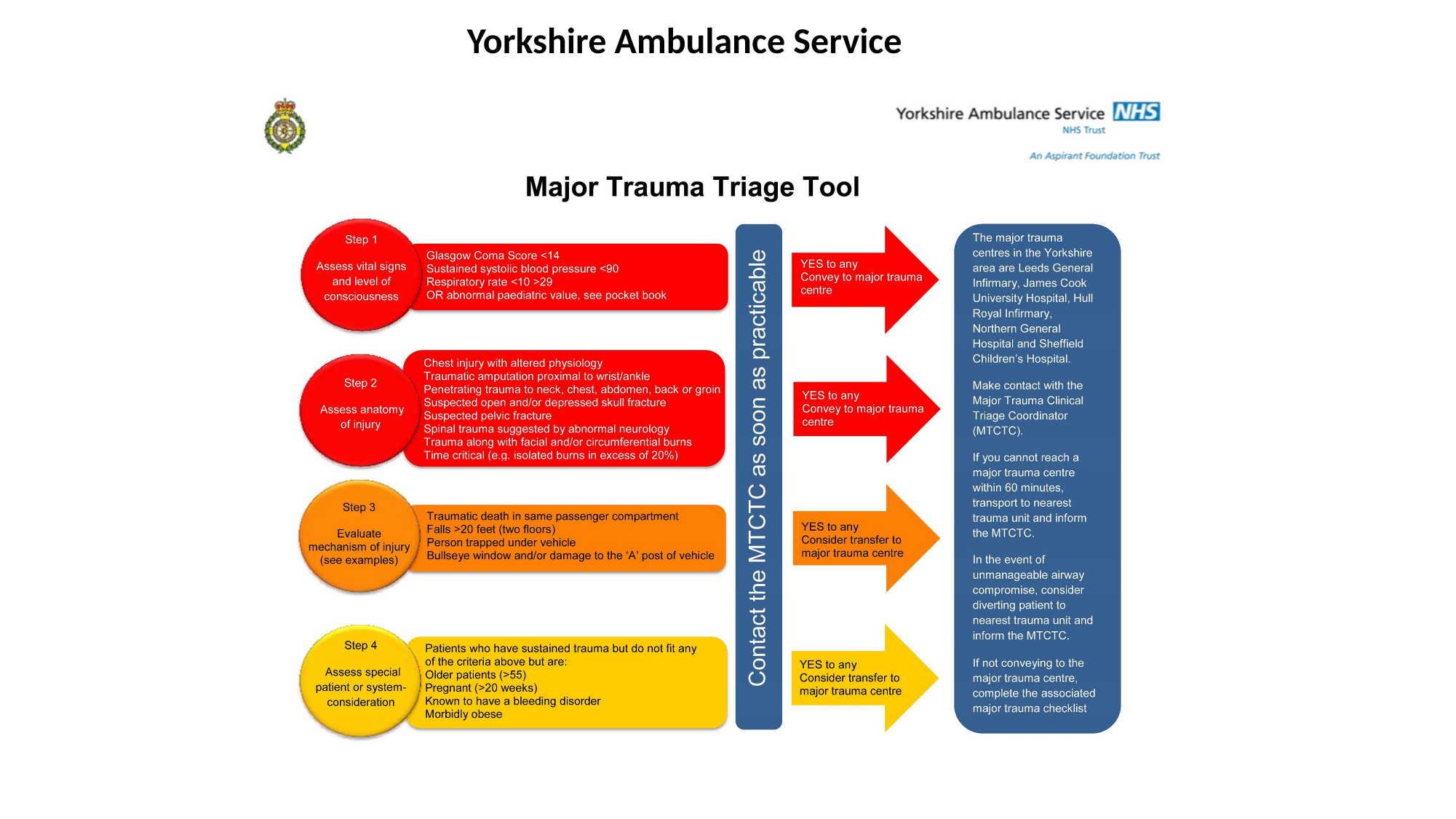

Yorkshire Ambulance Service

## Slide 3
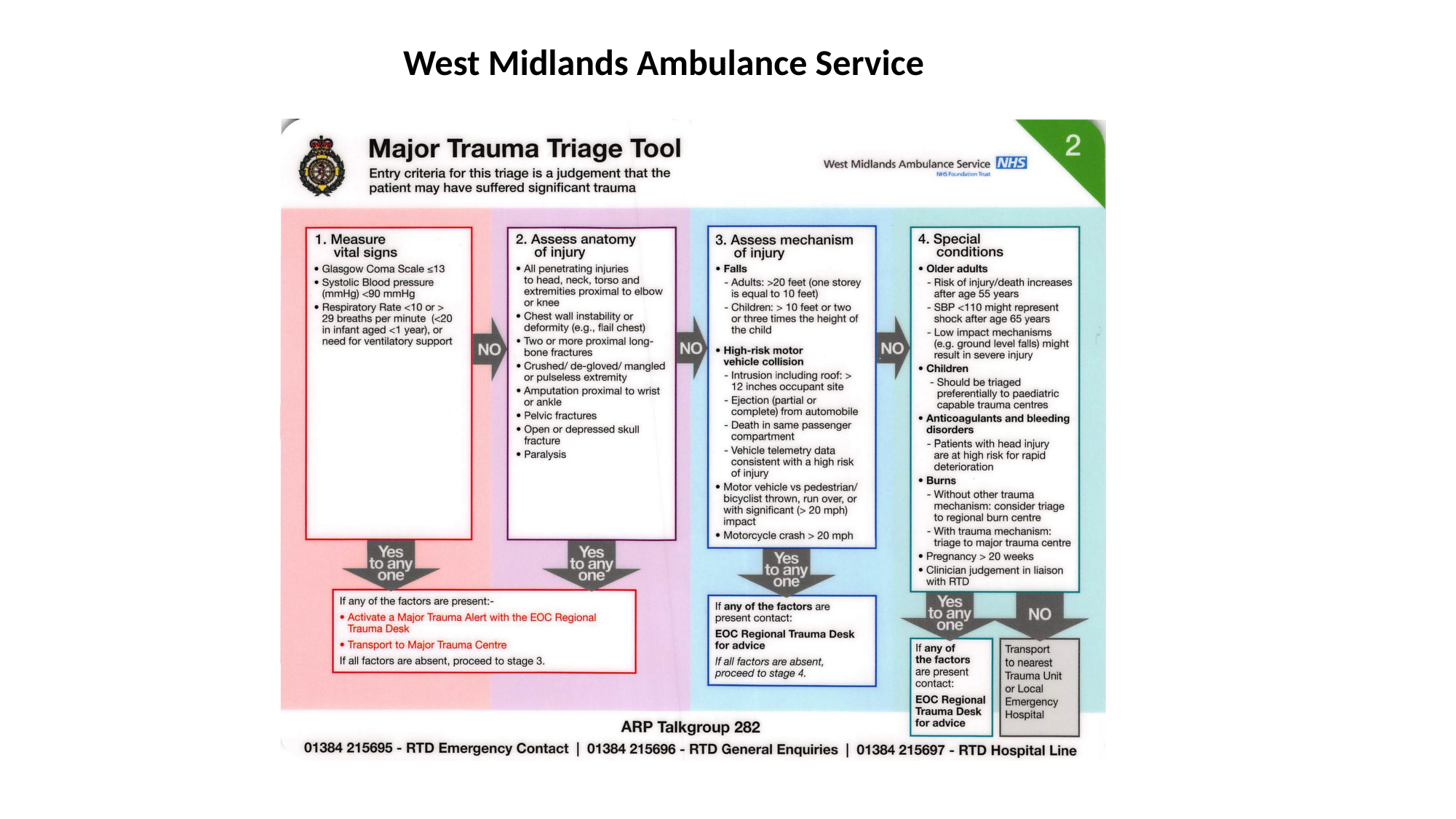

West Midlands Ambulance Service

## Slide 4
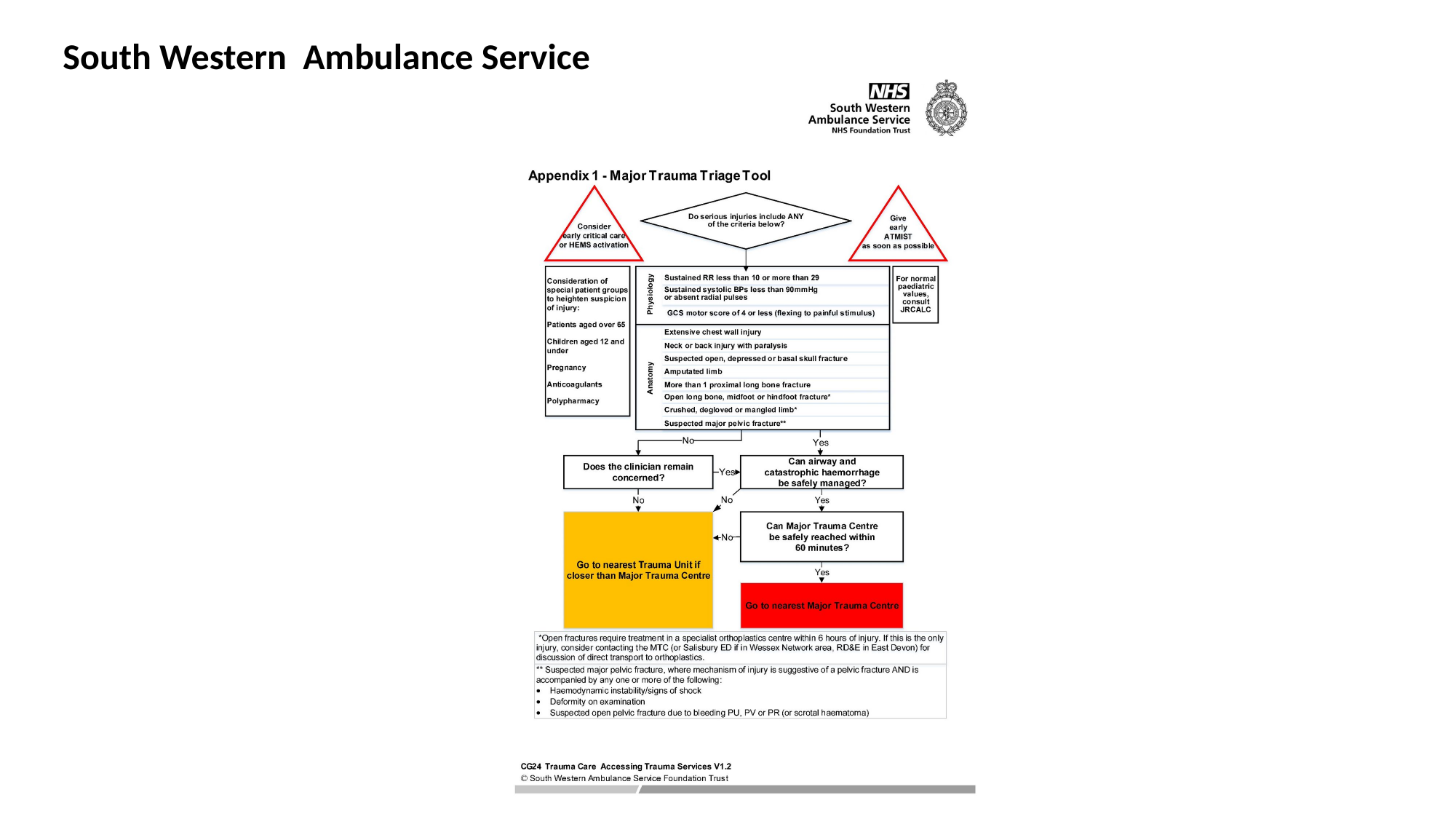

South Western Ambulance Service
