# Supplementary material for: The accuracy of prehospital triage decisions in English trauma networks – a case-cohort study
Source: Scand J Trauma Resusc Emerg Med. 2024 May 21;32:47. doi: 10.1186/s13049-024-01219-9 (PMC11110388; doi:10.1186/s13049-024-01219-9)

a) Natural frequencies bar plot for 1000 non-trivial injury patients

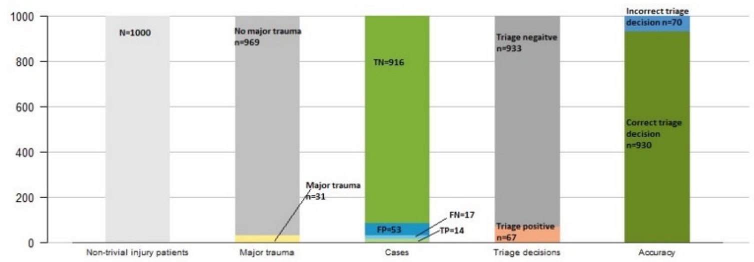

b) Prism plot showing natural frequencies and probabilities for 1000 non-trivial injury patients.

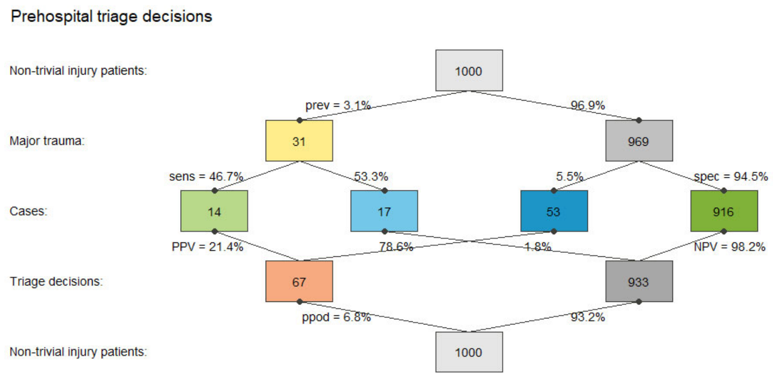

c) **Icon array classifying results for hypothetical 1000 individual population**

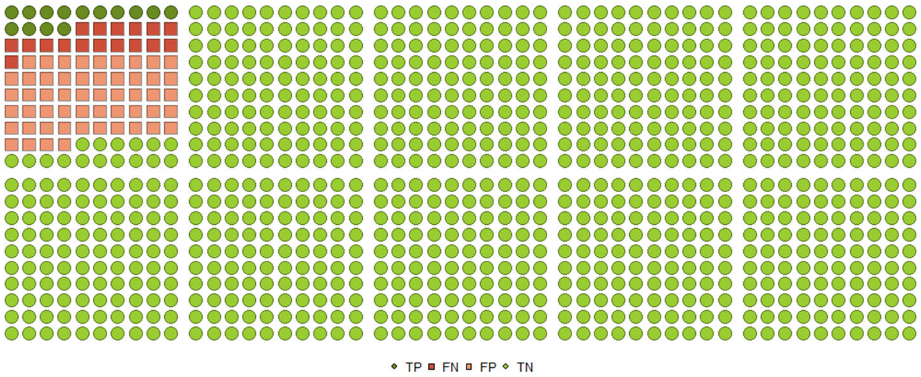

d) Fagan's nomogram showing pre- and post-test probabilities

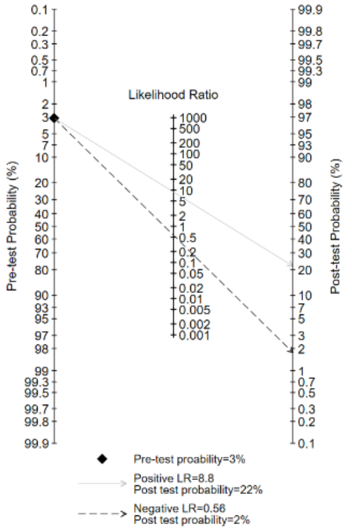

Supplement: Supplementary file 3 — Supplementary Material 3. [file 13049_2024_1219_MOESM3_ESM.pdf]
